# Resultados Clínicos após Implante de Marca-Passo: Um Estudo Comparativo de 3 Anos entre Síndrome do Nó Sinusal e Bloqueio Atrioventricular

**DOI:** 10.36660/abc.20250751

**Published:** 2026-05-20

**Authors:** Fuat Polat, Haşim Tüner, Veysi Can, Sait Terzi

**Affiliations:** 1 Department of Cardiology Dr. Siyami Ersek Thoracic and Cardiovascular Surgery Education Research Hospital Istanbul Turquia Department of Cardiology, Dr. Siyami Ersek Thoracic and Cardiovascular Surgery Education Research Hospital, Istanbul – Turquia; 2 Department of Cardiology Istanbul Arel University Memorial Bahcelievler Hospital Istanbul Turquia Department of Cardiology, Istanbul Arel University Memorial Bahcelievler Hospital, Istanbul – Turquia; 3 Department of Cardiology Van Health Sciences University Training and Research Hospital Van Turquia Department of Cardiology, Van Health Sciences University Training and Research Hospital, Van – Turquia; 4 Health Science University Istanbul Turquia Health Science University, Istanbul – Turquia

**Keywords:** Marca-Passo Artificial, Síndrome do Nó Sinusal, Bloqueio Atrioventricular, Fibrilação Atrial

## Abstract

**Fundamento:**

O implante de marca-passo permanente é um tratamento estabelecido tanto para a síndrome do nó sinusal (SNS) quanto para o bloqueio atrioventricular (BAV), porém existem poucos dados comparando os resultados clínicos a longo prazo e os padrões de remodelamento cardíaco entre essas distintas etiologias bradiarrítmicas.

**Objetivos:**

Comparar os resultados clínicos e as alterações estruturais cardíacas em três anos entre pacientes com SNS e BAV após implante de marca-passo permanente.

**Métodos:**

Este estudo observacional retrospectivo incluiu 192 pacientes adultos (65 com SNS, 127 com BAV) submetidos a implante de marca-passo entre janeiro de 2018 e dezembro de 2020. Dados demográficos, parâmetros ecocardiográficos, dados registrados pelo marca-passo e desfechos clínicos foram avaliados no início do estudo, após um mês, seis meses, um ano e três anos. Os desfechos primários incluíram alterações na estrutura e função cardíacas, melhora dos sintomas, fibrilação atrial de início recente, reinternação e mortalidade por todas as causas.

**Resultados:**

Ambos os grupos demonstraram declínios significativos na fração de ejeção do ventrículo esquerdo ao longo de três anos (SNS: 57,2±6,0% para 54,3±8,3%, p<0,001; BAV: 55,5±7,0% para 53,0±6,8%, p<0,001). Apesar da progressão semelhante da disfunção ventricular, padrões distintos de remodelamento emergiram: os pacientes com SNS apresentaram aumentos significativos no diâmetro do átrio esquerdo (31,3 para 38,8 mm, p=0,040), no diâmetro diastólico final do ventrículo esquerdo (45,2 para 51,0 mm, p<0,001) e na prevalência de regurgitação mitral (26,2% para 64,6%), enquanto os pacientes com BAV mantiveram dimensões ventriculares estáveis apesar da alta carga de estimulação ventricular (>90%). A melhora dos sintomas foi comparável (SNS: 85,6% vs. BAV: 83,5%, p=0,84), assim como o surgimento de fibrilação atrial (41,5% vs. 37,0%, p=0,54), a reinternação (50,8% vs. 58,3%, p=0,38) e a mortalidade por todas as causas (13,8% vs. 11,8%, p=0,69). Os preditores de mortalidade diferiram: gravidade da regurgitação mitral em pacientes com SNS versus doença arterial coronariana, terapia com betabloqueadores e volume do átrio esquerdo em pacientes com BAV.

**Conclusões:**

Apesar dos benefícios sintomáticos e resultados clínicos semelhantes, pacientes com SNS e BAV demonstram padrões distintos de remodelamento cardíaco e diferentes preditores de mortalidade, o que reforça a necessidade de estratégias de acompanhamento específicas para cada etiologia em pacientes com marca-passo.

## Graphical abstract

**Figura Central:**
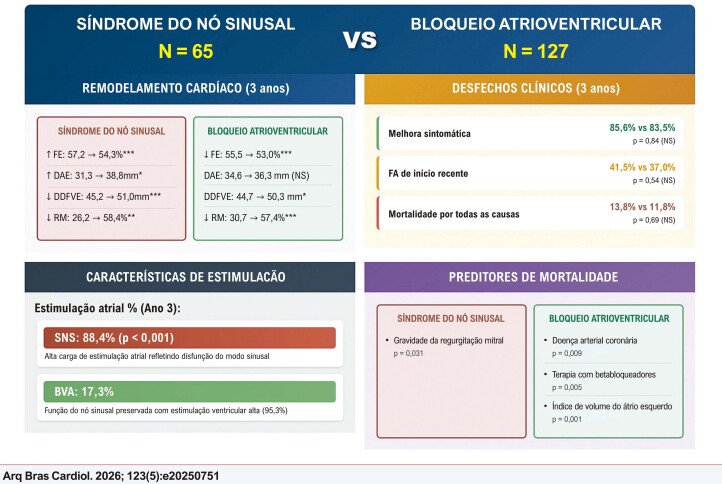
Resultados Clínicos após Implante de Marca-Passo: Um Estudo Comparativo de 3 Anos entre Síndrome do Nó Sinusal e Bloqueio Atrioventricular

Significância estatística: * p < 0,05; ** p < 0,01; *** p < 0,001; NS indica insignificância (p ≥ 0,05). Todas as comparações baseadas na linha de base vs. medições de acompanhamento de 3 anos. Código de cores: Caixas vermelhas/rosas: Resultados do grupo SNS; Caixas verdes: Resultados do grupo BAV; Seção laranja: Resultados clínicos; Seção roxa: Preditor de morbilidade. AVB: bloqueio atrioventricular; EF: Fração de ejeção; DDFVE: Diâmetro diastólico final do ventrículo esquerdo; RM: Regurgitação mitral; AF: fibrilação atrial; NS: insignificante. Setas: ↓ indica diminuição do valor basal para o acompanhamento de 3 anos; ↑ indica aumento do valor basal para o acompanhamento de 3 anos.

## Introdução

As bradicardias, incluindo a síndrome do nó sinusal (SNS) e o bloqueio atrioventricular (BAV), são indicações comuns para o implante de marca-passo permanente.^1^ Embora os marca-passos restaurem eficazmente a frequência cardíaca e previnam a síncope, seus efeitos a longo prazo na estrutura cardíaca, na função e nos desfechos clínicos ainda são objeto de investigação contínua.^2^ Compreender as diferenças na progressão da doença e na resposta à terapia de estimulação cardíaca entre pacientes com SNS e BAV é essencial para otimizar o manejo do paciente e melhorar os resultados prognósticos.^3^

A SNS é caracterizada por disfunção do nó sinusal, levando à bradicardia inadequada e sintomas como tontura, síncope e fadiga.^4^ Em contraste, o BAV resulta da condução prejudicada entre os átrios e os ventrículos, podendo levar à bradicardia grave e instabilidade hemodinâmica.^5^ Embora a terapia com marca-passo alivie eficazmente os sintomas em ambas as condições, persistem preocupações quanto ao seu impacto na remodelamento cardíaco, na função valvar e no desenvolvimento de insuficiência cardíaca ao longo do tempo.^6^

Estudos anteriores sugeriram que a estimulação do ventrículo direito pode contribuir para a remodelamento cardíaco adverso, incluindo disfunção do ventrículo esquerdo e aumento atrial, potencialmente aumentando o risco de fibrilação atrial (FA) e insuficiência cardíaca.^7^ No entanto, ainda não está claro se esses efeitos diferem significativamente entre pacientes com SNS e BAV.^8^ Além disso, as implicações clínicas da terapia com marca-passo, incluindo taxas de reinternação, mortalidade e melhora dos sintomas, requerem investigação adicional.^9^

Este estudo tem como objetivo comparar os resultados clínicos a longo prazo de pacientes submetidos a implante de marca-passo para SNS versus BAV durante um período de acompanhamento de três anos. Por meio da análise de alterações ecocardiográficas, dados registrados pelo marca-passo e desfechos clínicos, buscamos fornecer uma avaliação abrangente do impacto da terapia de estimulação cardíaca nessas duas populações distintas de pacientes. Os achados deste estudo contribuirão para uma melhor compreensão das implicações prognósticas do implante de marca-passo e orientarão futuras estratégias para o manejo do paciente e a otimização da terapia.

## Métodos

### Desenho do estudo e população

Este estudo foi um estudo de coorte observacional retrospectivo conduzido para comparar os desfechos clínicos a longo prazo de pacientes submetidos a implante de marca-passo permanente para SNS ou BAV. A população do estudo incluiu pacientes adultos submetidos a implante de marca-passo em nossa instituição entre janeiro de 2018 e dezembro de 2020. Os critérios de inclusão consistiram em diagnóstico confirmado de SNS ou BAV como indicação primária para implante de marca-passo, idade ≥ 18 anos e disponibilidade de dados clínicos e ecocardiográficos basais e de seguimento. Os critérios de exclusão incluíram implante prévio de marca-passo, doença valvar grave concomitante que exigisse intervenção, insuficiência cardíaca avançada (classe IV da *New York Heart Association [NYHA*]) ou histórico de cardiopatia congênita.

### Coleta e acompanhamento de dados

Dados demográficos, clínicos, laboratoriais e ecocardiográficos foram coletados de prontuários médicos. As características basais incluíram idade, sexo, índice de massa corporal (IMC), comorbidades (hipertensão, diabetes mellitus, doença arterial coronariana e tabagismo) e uso de medicamentos (betabloqueadores, inibidores da ECA/bloqueadores dos receptores da angiotensina [BRA], antagonistas dos receptores de mineralocorticoides [ARM], diuréticos de alça e bloqueadores dos canais de cálcio não diidropiridínicos). Os parâmetros laboratoriais incluíram sódio (Na) sérico, potássio (K), taxa de filtração glomerular estimada (TFGe) e níveis de hemoglobina.

### Avaliação ecocardiográfica

Todos os pacientes foram submetidos a ecocardiografia transtorácica no início do estudo e nos intervalos de acompanhamento (primeiro mês, sexto mês, primeiro ano e terceiro ano). Os exames ecocardiográficos foram realizados utilizando um sistema de ultrassom comercial (Philips EPIQ 7) por cardiologistas experientes, seguindo as diretrizes da Sociedade Americana de Ecocardiografia (ASE). Os principais parâmetros medidos foram os seguintes:

- *Fração de ejeção (FE) do ventrículo esquerdo:* Avaliado utilizando o método biplanar de Simpson a partir das vistas apical de quatro câmaras e de duas câmaras.
- *Diâmetro do átrio esquerdo (DAE):* Medido na vista paraesternal do eixo longo ao final da sístole.
- *Índice de Volume do Átrio Esquerdo (IVAE):* Calculado usando o método biplano área-comprimento e indexado à área da superfície corporal.
- *Diâmetro diastólico final do ventrículo esquerdo (DDFVE):* Medido ao nível das pontas das cúspides da válvula mitral na vista paraesternal do eixo longo.
- *Pressão sistólica da artéria pulmonar (PSAP):* Estimativa feita com base na velocidade máxima de regurgitação tricúspide e na estimativa da pressão atrial direita.
- *Gravidade da regurgitação mitral (RM):* A avaliação foi feita por meio de ecografia Doppler colorida e classificada como leve, moderada ou grave, com base na área do jato de regurgitação e na largura da vena contracta.

### Coleta e interpretação de dados de marca-passo

Os dados de programação do marca-passo foram obtidos a partir dos relatórios de verificação do dispositivo em cada consulta de acompanhamento. A verificação foi realizada utilizando o programador de marca-passo da Medtronic e envolveu a avaliação de:

- *Limite inferior médio da taxa:* A frequência cardíaca mínima programada na qual o marca-passo fornece suporte de estimulação.
- *Percentagem de estimulação atrial:* A proporção de tempo em que a estimulação atrial é administrada, indicando a dependência do marca-passo na estimulação artificial para manter o ritmo.
- *Percentagem de batimentos atriais prematuros:* A porcentagem de contrações atriais prematuras detectadas pode indicar uma arritmia atrial subjacente.
- *Episódios de Alta Frequência Atrial (EAFAs):* Episódios de atividade atrial rápida detectados pelo marca-passo, definidos como frequências atriais que excedem um limite predefinido (por exemplo, 180 batimentos por minuto), que podem sugerir FA ou taquiarritmia atrial.
- *Protocolo de Programação de Marca-passo:* Todos os marca-passos foram programados de acordo com as diretrizes atuais, com otimização para estimulação fisiológica. O modo de estimulação foi DDD/DDDR em todos os pacientes, com um limite inferior de frequência de 60 batimentos por minuto. Em pacientes com SNS, algoritmos para minimizar a estimulação ventricular foram ativados quando disponíveis, incluindo intervalo AV estendido (até 250-300 ms) e recursos de histerese AV para promover a condução AV intrínseca. No entanto, em pacientes com BAV, tais algoritmos não foram utilizados, dada a doença de condução intrínseca, e intervalos AV padrão (120-180 ms) foram programados. A estimulação responsiva à frequência foi ativada em pacientes com incompetência cronotrópica. Algoritmos de troca de modo para taquiarritmias atriais foram ativados em todos os dispositivos. Apesar dessas estratégias de programação, a carga de estimulação ventricular em pacientes com BAV permaneceu alta (>90%) devido à natureza de seu distúrbio de condução, enquanto pacientes com SNS mantiveram predominantemente a ativação ventricular intrínseca.

### Medidas de resultado

O desfecho primário foi a variação longitudinal dos parâmetros ecocardiográficos durante o período de acompanhamento de três anos. Os desfechos secundários incluíram:

- *Melhora dos sintomas, avaliada pela escore de sintomas da European Heart Rhythm Association (EHRA):* O escore EHRA é um sistema de classificação que avalia a gravidade dos sintomas relacionados à arritmia, variando de EHRA I (sem sintomas) a EHRA IV (sintomas graves que afetam as atividades diárias).
- *Incidência de FA de início recente:* Definida como o primeiro episódio documentado de FA detectado por eletrocardiograma de rotina, monitoramento Holter ou interrogatório de marca-passo, em um paciente sem histórico prévio de FA.
- *Taxas de reinternação durante o período de acompanhamento.*
- *Mortalidade por todas as causas.*

### Análise estatística

As variáveis contínuas foram expressas como média ± desvio padrão (DP) e comparadas entre os grupos utilizando o teste-t de Student para amostras independentes. As variáveis categóricas foram apresentadas como frequências e percentagens e analisadas utilizando o teste qui-quadrado ou o teste exato de Fisher. A análise de variância de medidas repetidas (ANOVA) foi utilizada para avaliar as alterações nos parâmetros ecocardiográficos ao longo do tempo. A análise de regressão de Cox com riscos proporcionais foi realizada para identificar preditores de reinternação e mortalidade. As curvas de sobrevida de Kaplan-Meier foram geradas para os desfechos de reinternação e mortalidade, e as comparações entre os grupos foram realizadas utilizando o teste de Log-Rank. Um valor de p bicaudal <0,05 foi considerado estatisticamente significativo.

### Considerações éticas

Este estudo foi conduzido de acordo com os princípios da Declaração de Helsinque e aprovado pelo comitê de ética institucional. Devido à natureza retrospectiva do estudo, o consentimento informado foi dispensado pelo comitê de ética. A confidencialidade dos pacientes foi mantida por meio da anonimização dos dados antes da análise.

## Resultado

O estudo incluiu um total de 192 pacientes, sendo que 65 (33,9%) foram submetidos a implante de marca-passo devido à SNS e 127 (66,1%) devido a BAV. A idade média da coorte foi de 66,3 ± 7,2 anos, e 89 pacientes (46,4%) eram do sexo masculino. A hipertensão foi a comorbidade mais prevalente, afetando 93 pacientes (48,4%).

Os dois grupos foram comparados em termos de características demográficas, ecocardiográficas e clínicas. Não foram observadas diferenças significativas em relação à idade, sexo ou IMC. A prevalência de hipertensão arterial, diabetes mellitus, doença arterial coronariana e tabagismo ativo foi semelhante entre os grupos; no entanto, a insuficiência cardíaca foi mais frequente em pacientes com BAV do que naqueles com SNS (17,3% vs. 10,8%, p=0,03). O uso de inibidores da ECA/BRA e ARM não diferiu entre os grupos. Contudo, betabloqueadores (69,2% vs. 13,4%, p<0,001) e bloqueadores dos canais de cálcio não diidropiridínicos (12,3% vs. 7,1%, p=0,01) foram prescritos com mais frequência em pacientes com SNS. Por outro lado, diuréticos de alça foram usados com mais frequência no grupo com BAV (20,5% vs. 9,2%, p=0,05). Os níveis séricos de sódio (Na), potássio (K), taxa de filtração glomerular estimada (TFGe) e hemoglobina foram comparáveis entre os dois grupos. Na avaliação ecocardiográfica, o DAE foi significativamente maior nos pacientes com bloqueio AV (34,6 ± 6,5 mm vs. 31,3 ± 6,9 mm, p=0,02), enquanto nenhuma outra diferença significativa foi observada nos demais parâmetros (Tabela 1).

**Tabela 1:** – Características dos pacientes, incluindo dados demográficos, ecocardiográficos e clínicos

Características do paciente	Todos os pacientes (N=192)	Pacientes com síndrome do nó sinusal (N=65)	Pacientes com bloqueio atrioventricular (N=127)	Valor de p
Idade (anos)	66,3±7,2	66,4±7,8	66,3±6,9	0,96
Sexo (masculino)	89 (46,4)	28 (43,1)	61 (48,0)	0,52
IMC (kg/m^2^)	27,4±4,4	27,7±4,8	27,2±4,2	0,43
Hipertensão	93 (48,4)	34 (52,3)	59 (46,5)	0,44
DM	34 (17,7)	12 (18,5)	22 (17,3)	0,85
Insuficiência cardíaca	29 (15,1)	7 (10,8)	22 (17,3)	0,03
Doença arterial coronária	59 (30,7)	25 (38,5)	34 (26,8)	0,10
Fumante ativo	94 (49,0)	34 (52,3)	60 (47,2)	0,32
Betabloqueador	62 (32,3)	45 (69,2)	17 (13,4)	<0,001
BCC não diidropiridínico	17 (8,9)	8 (12,3)	9 (7,1)	0,01
IECA/BRA	103 (53,6)	36 (55,4)	67 (52,8)	0,73
ARM	38 (19,8)	13 (20,0)	25 (19,7)	0,96
Diurético de alça	32 (16,7)	6 (9,2)	26 (20,5)	0,05
Na (mEq/L)	134,4±1,7	133,9±2,0	135,1±1,6	0,81
K (mmol/L)	4,3±1,1	4,4±1,0	4,3±1,2	0,89
TFG (mL/min/1,73m^2^)	91,3±18,7	91,6±18,5	91,1±18,9	0,84
Hemoglobina (mg/dL)	13,7±1,9	13,6±2,0	13,7±1,8	0,85
EF%	56,1±6,7	57,2±6,0	55,5±7,0	0,11
DAE	33,5±6,8	31,3±6,9	34,6±6,5	0,02
IVAE	45,6±9,7	46,1±10,8	45,3±9,1	0,58
DDFVE	44,9±3,4	45,2±3,5	44,7±3,4	0,28
IM	56 (29,1)	17 (26,2)	39 (30,7)	0,66
PASP	26,2±6,7	26,9±6,2	25,8±7,0	0,30

IECA/BRA: Inibidores da enzima conversora de angiotensina/Bloqueadores do receptor de angiotensina II; IMC: Índice de massa corporal; BCC: Bloqueador dos canais de cálcio; DM: Diabetes mellitus; FE: Fração de ejeção; TFG: Taxa de filtração glomerular; K: Potássio; DAE: Diâmetro do átrio esquerdo; IVAE: Índice de volume do átrio esquerdo; DDFVE: Diâmetro diastólico final do ventrículo esquerdo; RM: Regurgitação mitral; ARM: Antagonista do receptor de mineralocorticoides; Na: Sódio; PASP: Pressão sistólica da artéria pulmonar. *Os valores são média ± DP, mediana (IIQ) ou n [n/N se dados ausentes] (%). *Intervalo interquartil [percentil 25-percentil 75].

Os dois grupos de pacientes foram comparados utilizando dados registrados por marca-passo. O limite inferior médio da frequência cardíaca na linha de base não diferiu significativamente entre os grupos (60,5 ± 2,5 vs. 61,2 ± 3,6, p = 0,20). Como esperado, a porcentagem de estimulação atrial foi consistentemente maior no grupo com SNS em todos os intervalos de acompanhamento (primeiro mês: 84,5 ± 19,1% vs. 18,1 ± 14,5%; terceiro ano: 88,4 ± 20,3% vs. 17,3 ± 21,3%; p < 0,001), enquanto a porcentagem de estimulação ventricular foi marcadamente maior nos pacientes com bloqueio AV (primeiro mês: 92,5 ± 6,3% vs. 12,3 ± 8,5%; terceiro ano: 95,3 ± 4,8% vs. 9,8 ± 7,6%; p < 0,001). A estimulação ventricular mínima em pacientes com SNS reflete a preservação da condução AV nessa população, enquanto a alta carga de estimulação ventricular em pacientes com BAV foi inerente ao seu distúrbio de condução. A porcentagem de extrassístoles atriais e a frequência de *EAFAs* foram comparáveis entre os grupos ao longo do período de acompanhamento (Tabela 2).

**Tabela 2:** – Comparação dos dados do marca-passo entre os grupos de pacientes durante o período de acompanhamento

Dados do marca-passo	Pacientes com síndrome do nó sinusal	Pacientes com bloqueio atrioventricular	Valor de p
**Limite inferior médio de frequência do marca-passo (bpm)**	60,5±2,5	61,2±3,6	0,20
**Percentagem de estimulação atrial (%)**			
Primeiro mês	84,5±19,1	18,1±14,5	<0,001
Sexto mês	86,0±19,4	15,2±20,0	<0,001
Primeiro ano	87,2±22,2	16,8±22,6	<0,001
Terceiro ano	88,4±20,3	17,3±21,3	<0,001
**Estimulação ventricular (%)**			
Primeiro mês	12,3±8,5	92,5±6,3	<0,001
Sexto mês	11,8±9,2	93,1±5,8	<0,001
Primeiro ano	10,5±8,9	94,2±5,1	<0,001
Terceiro ano	9,8±7,6	95,3±4,8	<0,001
**Percentagem de batimentos atriais prematuros (%)**			
Primeiro mês	5,9±2,1	6,4±1,9	0,57
Sexto mês	5,9±1,7	6,3±1,4	0,66
Primeiro ano	4,1±1,5	3,7±1,2	0,45
Terceiro ano	3,0±1,1	2,9±1,1	0,77
**Episódio de alta frequência atrial (%)**			
Primeiro mês	9,9±1,8	10,3±4,1	0,88
Sexto mês	9,9±3,9	10,1±3,2	0,97
Primeiro ano	14±3,2	13,9±4,3	0,98
Terceiro ano	17,2±1,6	15,1±2,9	0,70

bpm: Batimentos por minuto. *Os valores são média ± DP, mediana (IIQ) ou n [n/N se dados ausentes] (%). *Intervalo interquartil [percentil 25-percentil 75].

As alterações longitudinais nos parâmetros ecocardiográficos foram avaliadas desde o período pré-implante até o acompanhamento de 3 anos em ambos os subgrupos de pacientes. Entre os pacientes com SNS, observamos deterioração significativa em múltiplos parâmetros cardíacos. A função sistólica do ventrículo esquerdo diminuiu, com a FE reduzindo de 57,2 ± 6,0% no início do estudo para 54,3 ± 8,3% em 3 anos (p < 0,001). Esses pacientes apresentaram remodelamento atrial esquerdo progressivo, demonstrado pelo aumento do DAE de 31,3 ± 6,9 mm para 38,8 ± 7,9 mm (p = 0,040) e do IVAE de 46,1 ± 10,8 mL/m^2^ para 53,2 ± 10,4 mL/m^2^ (p < 0,001). Além disso, as dimensões do ventrículo esquerdo aumentaram significativamente, com o DDFVE aumentando de 45,2 ± 3,5 mm para 51,0 ± 3,5 mm (p < 0,001). A função valvar também piorou, com a prevalência de RM leve ou grave aumentando substancialmente de 26,2% (17/65 pacientes) para 58,4% (38/65 pacientes; p = 0,001). Adicionalmente, a PSAP aumentou de 26,9 ± 6,2 mmHg para 31,6 ± 7,0 mmHg (p = 0,025). Pacientes com BAV também demonstraram uma redução significativa na FE de 55,5 ± 7,0% para 53,0 ± 6,8% (p < 0,001). No entanto, ao contrário do grupo SNS, os pacientes com BAV mantiveram dimensões estáveis do átrio esquerdo (DAE e IVAE) e do DDFVE durante todo o período de acompanhamento. Ambos os grupos apresentaram progressão comparável da disfunção valvar, com os pacientes com BAV exibindo um aumento na RM leve ou grave de 30,7% (39/127 pacientes) para 57,4% (73/127 pacientes; p < 0,001) e um aumento na PASP de 25,8 ± 7,0 mmHg para 30,8 ± 8,9 mmHg (p = 0,019) (Tabela 3).

**Tabela 3:** – Comparação dos dados ecocardiográficos entre os grupos de pacientes no início do estudo e após 3 anos de acompanhamento

Dados ecocardiográficos	Síndrome do nó sinusal			Bloqueio atrioventricular		
	Antes de MP	3º ano após o MP	Valor de p	Antes de MP	3º ano após o primeiro-ministro	Valor de p
FE%	57,2±6,0	54,3±8,3	<0,001	55,5±7,0	53,0±6,8	<0,001
DAE	31,3±6,9	38,8±7,9	0,040	34,6±6,5	36,3±7,4	0,10
IVAE	46,1±10,8	53,2±10,4	<0,001	45,3±9,1	48,4±9,2	0,14
LVEDD	45,2±3,5	51,0±3,5	<0,001	44,7±3,4	50,3±3,2	0,048
RM	17 (26,2)	38 (58,4)	0,001	39 (30,7)	73 (57,4)	<0,001
PASP	26,9±6,2	31,6±7,0	0,025	25,8±7,0	30,8±8,9	0,019

FE: Fração de ejeção; DAE: Diâmetro do átrio esquerdo; IVAE: Índice de volume do átrio esquerdo; DDFVE: Diâmetro diastólico final do ventrículo esquerdo; RM: Regurgitação mitral; PASP: Pressão sistólica da artéria pulmonar. *Os valores são média ± DP; mediana (intervalo interquartil) ou n [n/N se dados ausentes] (%). *Intervalo interquartil [percentil 25-percentil 75].

**Figura 1:**
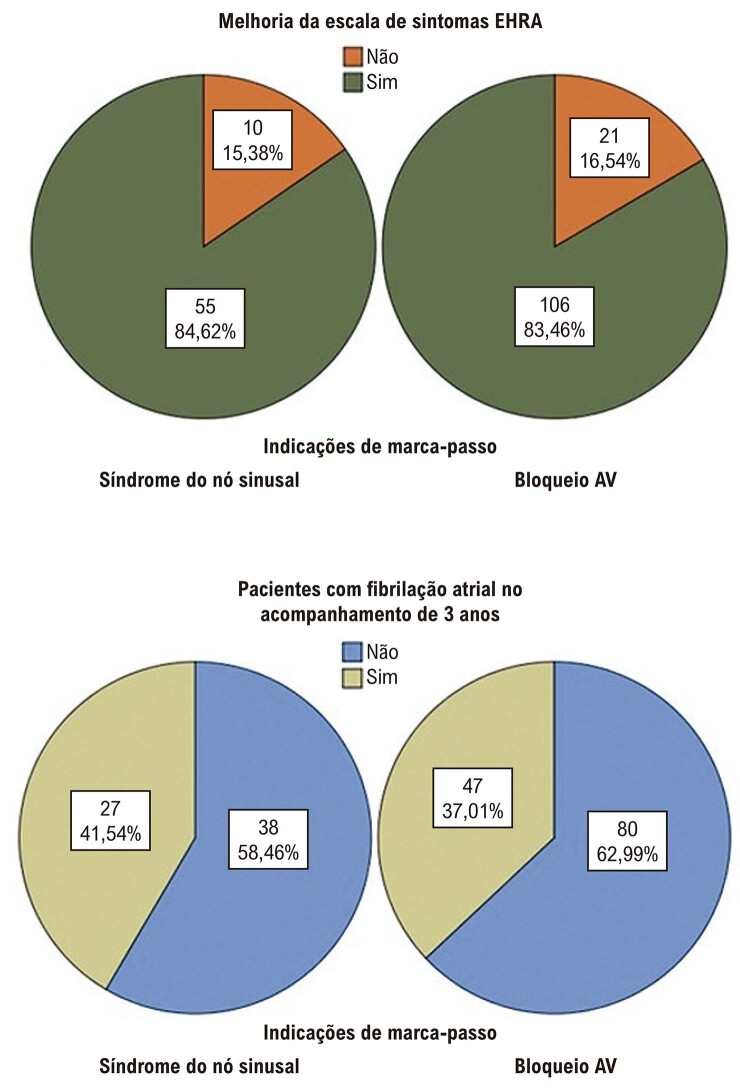
– Comparação da escala de sintomas EHRA e desenvolvimento de fibrilação atrial entre grupos de pacientes após 3 anos de acompanhamento.

Na avaliação de acompanhamento de 3 anos, os resultados clínicos foram avaliados de acordo com os escores de sintomas da EHRA. A análise revelou que 83,9% dos pacientes (161 de 192) apresentaram melhora sintomática após o implante de marca-passo. Quando estratificados por indicação, 85,6% dos pacientes com SNS (55 de 65) e 83,5% daqueles com BAV (106 de 127) demonstraram benefício clínico, sem diferença estatisticamente significativa entre os grupos (p=0,84; odds ratio [OR] 0,92, intervalo de confiança [IC] de 95% 0,40–2,09). A análise subsequente das características dos pacientes associadas à melhora sintomática revelou padrões distintos para cada grupo. Entre os pacientes com SNS, não foram identificados preditores significativos de resposta ao tratamento. Contudo, na coorte com BAV, diversos fatores apresentaram correlações inversas significativas com o benefício sintomático: sexo feminino (p=0,039), doença arterial coronariana preexistente (p=0,006), aumento do DAE (p=0,05) e maior DDFVE (p=0,042). Durante o período de observação de 3 anos, FA de início recente foi documentada em 38,5% da população do estudo (74 de 192). A incidência foi comparável entre os grupos etiológicos, ocorrendo em 41,5% dos pacientes com SNS (27 de 65) versus 37,0% dos pacientes com BAV (47 de 127) (p=0,54; OR 0,83, IC 95% 0,45–1,52). A análise de potenciais fatores de risco para o desenvolvimento de FA não revelou associações significativas no grupo com SNS. Por outro lado, entre os pacientes com BAV, três parâmetros emergiram como preditores significativos da ocorrência de FA: idade avançada (p=0,045), maior DAE (p=0,003) e aumento do IVAE (p=0,046) (Figura 1).

Na avaliação de acompanhamento de 3 anos, avaliamos os desfechos de reinternação e mortalidade em toda a coorte do estudo. Entre os 192 pacientes incluídos, 107 (55,7%) necessitaram de reinternação durante o período de observação. Quando estratificadas pela indicação do marca-passo, as taxas de reinternação foram comparáveis entre os grupos, ocorrendo em 50,8% dos pacientes com SNS (33/65) versus 58,3% dos pacientes com BAV (74/127) (p=0,38; odds ratio [OR] 1,20, intervalo de confiança [IC] de 95% 0,80-1,81).

**Figura 2:**
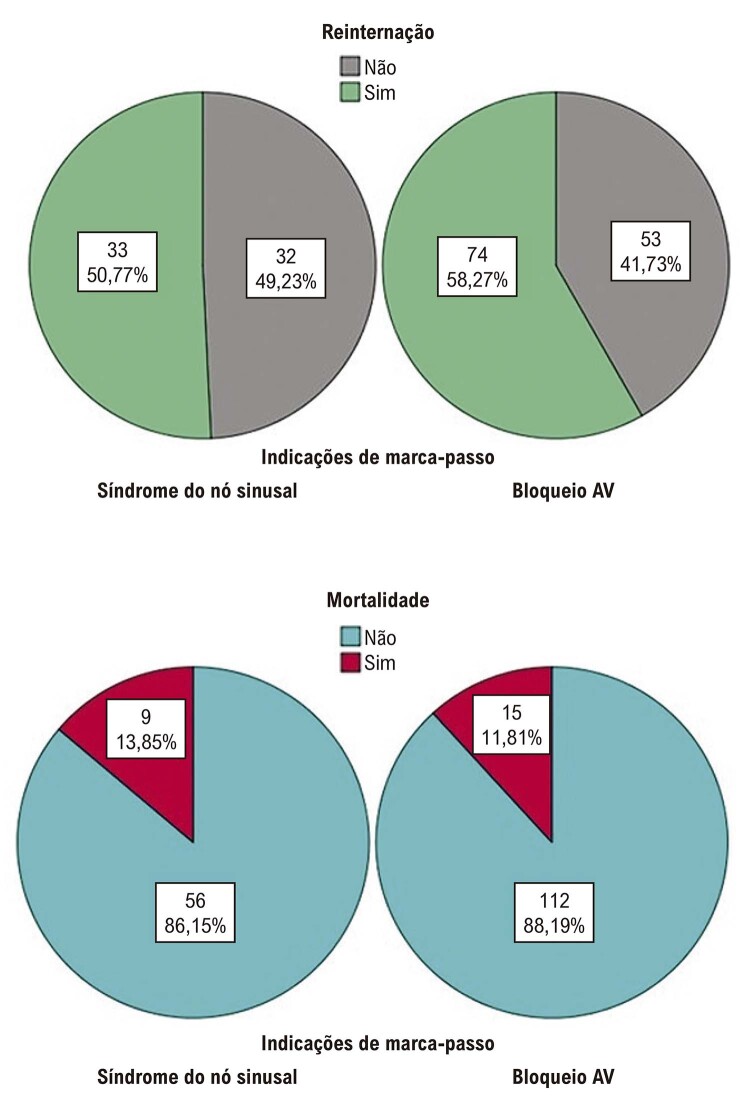
– Comparação de eventos de reinternação e mortalidade entre grupos de pacientes após 3 anos de acompanhamento.

A análise preditiva dos fatores de risco de reinternação revelou padrões distintos entre as etiologias. Para pacientes com SNS, apenas a PSAP elevada demonstrou associação significativa com reinternação (p=0,04). Em contraste, a coorte com BAV apresentou relações inversas significativas entre reinternação e sexo feminino (p=0,037) e diagnóstico de hipertensão (p=0,013). A mortalidade por todas as causas foi documentada em 12,5% dos pacientes (24/192), sem diferença significativa entre os grupos (SNS: 13,8% [9/65] vs. BAV: 11,8% [15/127]; p=0,69; OR 0,84, IC 95% 0,37-1,93). Os preditores de mortalidade diferiram substancialmente entre os grupos. Entre os pacientes com SNS, apenas a gravidade da RM apresentou correlação significativa com o risco de mortalidade (p=0,031). Por outro lado, os pacientes com BAV demonstraram associações significativas de mortalidade com três fatores independentes: doença arterial coronariana pré-existente (p=0,009), terapia com betabloqueadores (p=0,005) e IVAE (p=0,001) (Figura 2).

**Figura 3:**
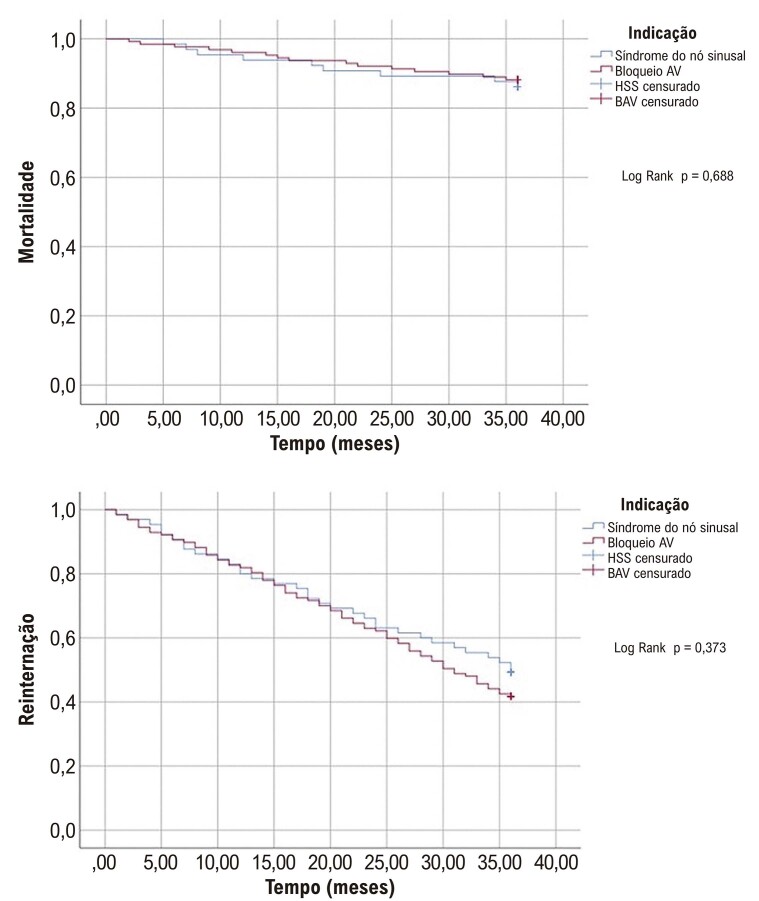
– Análise de Log-Rank de eventos de reinternação e mortalidade em um acompanhamento de 3 anos.

Durante o acompanhamento de 3 anos, os grupos foram comparados utilizando regressão de Cox e análise de sobrevida para os desfechos de reinternação e mortalidade. Não foi observada diferença significativa na mortalidade entre os grupos (Log-Rank p=0,688). No entanto, a análise de sobrevida para reinternação revelou uma tendência a menores taxas de reinternação ao longo do tempo em pacientes com SNS, embora essa diferença não tenha atingido significância estatística (Log-Rank p=0,373) (Figura 3). Os principais achados comparativos entre os grupos com SNS e BAV em relação à remodelamento cardíaco, desfechos clínicos, características de estimulação e preditores de mortalidade estão resumidos na Ilustração Central.

## Discussão

Este estudo fornece uma comparação abrangente dos resultados clínicos a longo prazo entre pacientes com SNS e BAV após implante de marca-passo permanente. Nossos achados demonstram que, embora a terapia com marca-passo proporcione melhora sintomática na maioria dos pacientes de ambos os grupos, existem diferenças importantes nos padrões de remodelamento cardíaco e nos preditores de desfechos adversos.

A maior porcentagem de estimulação atrial observada em pacientes com SNS é consistente com a disfunção subjacente do nó sinusal, que exige suporte de estimulação atrial mais frequente.^4,10^ Essa descoberta está alinhada com estudos anteriores que demonstraram a dependência de pacientes com SNS em relação à estimulação atrial para manter frequências cardíacas adequadas.^11^ Em contraste, pacientes com BAV tipicamente mantêm a função intrínseca do nó sinusal, mas necessitam de estimulação ventricular devido à condução AV prejudicada.^5^

As diferenças substanciais na carga de estimulação ventricular entre os grupos (>90% em pacientes com BAV vs. <12% em pacientes com SNS) fornecem um contexto crucial para a interpretação dos nossos achados de remodelamento. A alta porcentagem de estimulação ventricular em pacientes com BAV (média de 93-95% durante o acompanhamento) provavelmente contribuiu para os efeitos adversos observados em ambos os grupos, embora os padrões tenham diferido. Enquanto ambos os grupos apresentaram redução da FE, a dilatação progressiva do átrio e ventrículo esquerdos observada predominantemente em pacientes com SNS, apesar da estimulação ventricular mínima, sugere que a disfunção do nó sinusal em si pode predispor ao remodelamento adverso, independentemente da carga de estimulação. Por outro lado, as dimensões estáveis das câmaras em pacientes com BAV, apesar da estimulação ventricular quase universal, desafiam a noção de que a estimulação do ventrículo direito causa universalmente dilatação das câmaras, sugerindo que outros fatores, como doença atrial intrínseca na SNS, podem desempenhar papéis importantes. Esses achados estão em consonância com observações recentes de que os efeitos deletérios da estimulação do VD podem ser mais pronunciados em pacientes com patologia atrial subjacente.

Uma descoberta particularmente notável foi o padrão diferencial de remodelamento cardíaco entre os dois grupos. Os pacientes com SNS apresentaram aumentos significativos nas dimensões do átrio esquerdo e no DDFVE durante o período de acompanhamento, sugerindo remodelamento cardíaco adverso progressivo. Essa observação corrobora estudos anteriores que sugerem que a estimulação do ventrículo direito pode contribuir para a dissincronia ventricular e subsequente remodelamento adverso.^7,12^ A manutenção de dimensões estáveis do átrio esquerdo em pacientes com BAV, apesar de reduções comparáveis na FE, sugere potenciais diferenças na resposta fisiopatológica à terapia de estimulação cardíaca entre essas duas condições.

A incidência substancial de FA de início recente em ambos os grupos (41,5% no grupo com SNS vs. 37,0% no grupo com BAV) destaca a estreita relação entre bradiarritmia e o desenvolvimento de FA.^13^ Embora a diferença entre os grupos não tenha sido estatisticamente significativa, a identificação da idade avançada e do aumento das dimensões do átrio esquerdo como preditores de FA em pacientes com BAV está em consonância com os fatores de risco já estabelecidos para essa arritmia.^14^ A alta incidência de FA levanta questões importantes sobre os potenciais benefícios da anticoagulação profilática em pacientes de alto risco com marca-passo, particularmente aqueles com aumento das dimensões do átrio esquerdo.^15^

Em relação à melhora dos sintomas, nossa descoberta de que aproximadamente 84% dos pacientes apresentaram benefício clínico após o implante de marca-passo confirma a eficácia da terapia de estimulação cardíaca para o alívio dos sintomas em bradiarritmias.^16^ A ausência de diferenças significativas entre os grupos na melhora dos sintomas sugere que a etiologia subjacente da bradiarritmia pode não influenciar substancialmente a resposta subjetiva à terapia de estimulação cardíaca. No entanto, a identificação do sexo feminino, da doença arterial coronariana e do aumento das dimensões cardíacas como preditores negativos de melhora dos sintomas em pacientes com BAV justifica uma investigação mais aprofundada e pode ter implicações para a seleção e o aconselhamento de pacientes.^17^

A taxa de reinternação de aproximadamente 56% ao longo de três anos indica um ônus substancial para o sistema de saúde associado a pacientes com marca-passo. Embora a tendência para taxas de reinternação mais baixas em pacientes com SNS não tenha atingido significância estatística, os diferentes preditores de reinternação entre os grupos sugerem mecanismos fisiopatológicos potencialmente distintos que contribuem para a descompensação clínica.^18^ A associação entre pressão sistólica elevada na artéria pulmonar e reinternação em pacientes com SNS aponta para a potencial importância da função ventricular direita e da hemodinâmica pulmonar nessa população.^19^

Os desfechos de mortalidade foram comparáveis entre os grupos, afetando aproximadamente 12% dos pacientes ao longo de três anos. No entanto, os distintos preditores de mortalidade — gravidade da RM em pacientes com SNS versus doença arterial coronariana, terapia com betabloqueadores e aumento do volume do átrio esquerdo em pacientes com BAV — sugerem diferentes vias que contribuem para desfechos adversos.^20,21^ A associação entre terapia com betabloqueadores e mortalidade em pacientes com BAV é particularmente intrigante e pode refletir tanto a gravidade da doença cardíaca subjacente que exige o uso de betabloqueadores quanto interações potencialmente adversas entre a terapia farmacológica e o marca-passo.^22^

O aumento progressivo da RM observado em ambos os grupos merece atenção especial. Este achado é consistente com estudos anteriores que sugerem que a estimulação do ventrículo direito pode induzir dissincronia do músculo papilar e subsequente RM funcional.^23^ A correlação entre a gravidade da RM e a mortalidade em pacientes com SNS enfatiza a potencial importância prognóstica da função valvar nessa população.^24^

Este estudo apresenta diversos avanços importantes em relação à literatura existente. Primeiro, demonstramos pela primeira vez que pacientes com SNS exibem remodelamento cardíaco progressivo apesar da estimulação ventricular mínima (<12%), contradizendo a suposição predominante de que o remodelamento adverso é primariamente induzido pela estimulação. Essa descoberta sugere um processo fisiopatológico intrínseco na SNS que merece ser reconhecido como um fator de risco independente para deterioração estrutural, não enfatizado anteriormente em grandes estudos com marca-passos, como o MOST ou o DAVID. Segundo, nossa observação de que pacientes com BAV mantêm dimensões ventriculares estáveis apesar de >90% de estimulação ventricular questiona a universalidade da cardiomiopatia induzida por estimulação e sugere fatores protetores ou mecanismos compensatórios que merecem investigação adicional. Terceiro, identificamos preditores de mortalidade específicos da etiologia que não foram descritos anteriormente: enquanto a RM prediz desfechos na SNS (potencialmente relacionada à disfunção atrial progressiva), o valor preditivo do índice de volume atrial esquerdo em pacientes com BAV sugere vias mecanísticas diferentes para desfechos adversos. Esses perfis de risco distintos fornecem uma justificativa para estratégias de vigilância diferenciadas com base na etiologia subjacente da bradiarritmia — uma abordagem de medicina de precisão que atualmente não está refletida nas recomendações das diretrizes, que tratam todos os pacientes com marca-passo de forma semelhante, independentemente da indicação. Por fim, a alta incidência de FA de início recente (41,5% no grupo SNS e 37,0% no grupo BAV) excede substancialmente as taxas relatadas em estudos anteriores, provavelmente refletindo tanto a melhoria na detecção por meio do monitoramento contínuo do dispositivo quanto o nosso período de acompanhamento mais longo, o que enfatiza a necessidade de vigilância sistemática de arritmias atriais em todos os pacientes com marca-passo.

Nosso estudo apresenta diversas limitações que devem ser reconhecidas. Primeiro, seu desenho retrospectivo limita a inferência causal e pode introduzir viés de seleção. Segundo, não avaliamos sistematicamente a dissincronia ventricular, o que poderia ter fornecido informações mecanísticas sobre os padrões de remodelamento cardíaco observados. Terceiro, o fato de nosso estudo ter sido realizado em um único centro pode limitar a generalização dos resultados para outras populações. Por fim, não analisamos o impacto de diferentes modos ou configurações de estimulação cardíaca, o que pode influenciar os desfechos a longo prazo.

Apesar dessas limitações, nossos achados têm implicações clínicas importantes. Os diferentes padrões de remodelamento cardíaco e preditores de desfechos adversos entre pacientes com SNS e BAV sugerem que estratégias de acompanhamento individualizadas, baseadas na etiologia subjacente da bradiarritmia, podem ser benéficas. Pacientes com SNS podem se beneficiar de um monitoramento mais rigoroso das dimensões cardíacas e da otimização dos parâmetros de estimulação para minimizar a sobrecarga ventricular.^25^ Em pacientes com BAV, a atenção especial aos parâmetros do átrio esquerdo e ao manejo da doença arterial coronariana subjacente podem melhorar os desfechos a longo prazo.^26^

Pesquisas futuras devem explorar se estratégias alternativas de estimulação, como a estimulação do feixe de His ou a terapia de ressincronização cardíaca, podem prevenir as alterações estruturais observadas e melhorar os resultados em subgrupos específicos de pacientes.^27,28^ Além disso, são necessários estudos prospectivos com períodos de acompanhamento mais longos para melhor compreender o impacto a longo prazo da terapia de estimulação na estrutura e função cardíacas, particularmente em relação ao desenvolvimento de insuficiência cardíaca e disfunção valvar.^29^

## Conclusão

Este estudo comparativo de três anos demonstra que, embora o implante de marca-passo permanente proporcione melhora sintomática comparável em pacientes com SNS e BAV, as duas condições apresentam respostas fisiopatológicas fundamentalmente diferentes à terapia de estimulação cardíaca. Apesar da carga mínima de estimulação ventricular, os pacientes com SNS desenvolveram remodelamento cardíaco adverso progressivo com dilatação significativa das câmaras atrial e ventricular esquerdas, sugerindo que a disfunção do nó sinusal em si, independentemente da estimulação, predispõe à deterioração estrutural. Por outro lado, os pacientes com BAV mantiveram dimensões ventriculares estáveis apesar da estimulação ventricular quase universal (>90%), desafiando as suposições convencionais sobre os efeitos uniformemente deletérios da estimulação ventricular direita.

Os distintos preditores de mortalidade entre os grupos, a gravidade da RM na SNS versus doença arterial coronariana e o volume do átrio esquerdo no BAV reforçam a necessidade de estratificação de risco específica para cada etiologia e de estratégias de acompanhamento personalizadas. Esses achados corroboram abordagens de manejo individualizadas: vigilância ecocardiográfica mais rigorosa para detectar alterações estruturais em pacientes com SNS e maior atenção aos parâmetros do átrio esquerdo e ao manejo da doença coronariana em pacientes com BAV. Pesquisas futuras devem investigar se estratégias alternativas de estimulação cardíaca podem mitigar a tendência intrínseca de remodelamento observada em pacientes com SNS e validar protocolos de vigilância baseados em risco para cada população de pacientes.
