# Supplementary material for: Resultados Clínicos após Implante de Marca-Passo: Um Estudo Comparativo de 3 Anos entre Síndrome do Nó Sinusal e Bloqueio Atrioventricular
Source: Arq Bras Cardiol. 2026 May 20;123(5):e20250751. [Article in Portuguese] doi: 10.36660/abc.20250751 (PMC13399111; doi:10.36660/abc.20250751)
Supplement: Translation

# Translation: Clinical Outcomes Following PM Implantation: A 3-Year Comparative Study of Sick Sinus Syndrome and Atrioventricular Block

**DOI:** 10.36660/abc.20250751i

**Authors:** Fuat Polat, Haşim Tüner, Veysi Can, Sait Terzi

**Affiliations:** 1 Türkiye Department of Cardiology, Dr. Siyami Ersek Thoracic and Cardiovascular Surgery Education Research Hospital, Istanbul – Türkiye; 2 Türkiye Department of Cardiology, Istanbul Arel University Memorial Bahcelievler Hospital, Istanbul – Türkiye; 3 Türkiye Department of Cardiology, Van Health Sciences University Training and Research Hospital, Van – Türkiye; 4 Türkiye Health Science University, Istanbul – Türkiye

**Keywords:** Artificial Pacemaker, Sick Sinus Syndrome, Atrioventricular Block, Atrial Fibrillation

## Abstract

**Background:**

Permanent pacemaker implantation is an established treatment for both sick sinus syndrome (SSS) and atrioventricular block (AVB), yet limited data exist comparing long-term clinical outcomes and cardiac remodeling patterns between these distinct bradyarrhythmic etiologies.

**Objectives:**

To compare three-year clinical outcomes and cardiac structural changes between SSS and AVB patients following permanent pacemaker implantation.

**Methods:**

This retrospective observational study enrolled 192 adult patients (65 SSS, 127 AVB) who underwent pacemaker implantation between January 2018 and December 2020. Demographic data, echocardiographic parameters, pacemaker-recorded data, and clinical outcomes were assessed at baseline, one month, six months, one year, and three years. Primary outcomes included changes in cardiac structure and function, symptomatic improvement, new-onset atrial fibrillation, rehospitalization, and all-cause mortality.

**Results:**

Both groups demonstrated significant declines in left ventricular ejection fraction over three years (SSS: 57.2±6.0% to 54.3±8.3%, p<0.001; AVB: 55.5±7.0% to 53.0±6.8%, p<0.001). Despite similar ventricular dysfunction progression, distinct remodeling patterns emerged: SSS patients exhibited significant increases in left atrial diameter (31.3 to 38.8 mm, p=0.040), left ventricular end-diastolic diameter (45.2 to 51.0 mm, p<0.001), and mitral regurgitation prevalence (26.2% to 64.6%), while AVB patients maintained stable chamber dimensions despite high ventricular pacing burden (>90%). Symptomatic improvement was comparable (SSS: 85.6% vs. AVB: 83.5%, p=0.84), as were new-onset atrial fibrillation (41.5% vs. 37.0%, p=0.54), rehospitalization (50.8% vs. 58.3%, p=0.38), and all-cause mortality (13.8% vs. 11.8%, p=0.69). Mortality predictors differed: mitral regurgitation severity in SSS versus coronary artery disease, beta-blocker therapy, and left atrial volume in AVB patients.

**Conclusions:**

Despite similar symptomatic benefits and clinical outcomes, SSS and AVB patients demonstrate distinct cardiac remodeling patterns and different mortality predictors, supporting the need for etiology-specific follow-up strategies in pacemaker recipients.

## Introduction

Bradyarrhythmias, including sick sinus syndrome (SSS) and atrioventricular block (AVB), are common indications for permanent pacemaker implantation.^1^ While pacemakers effectively restore heart rate and prevent syncope, their long-term effects on cardiac structure, function, and clinical outcomes remain a subject of ongoing investigation.^2^ Understanding the differences in disease progression and response to pacing therapy between SSS and AVB patients is essential for optimizing patient management and improving prognostic outcomes.^3^

SSS is characterized by sinus node dysfunction, leading to inappropriate bradycardia and symptoms such as dizziness, syncope, and fatigue.^4^ In contrast, AVB results from impaired conduction between the atria and ventricles, potentially leading to severe bradycardia and hemodynamic instability.^5^ Although pacemaker therapy effectively alleviates symptoms in both conditions, concerns persist regarding its impact on cardiac remodelling, valvular function, and the development of heart failure over time.^6^

Previous studies have suggested that right ventricular pacing may contribute to adverse cardiac remodelling, including left ventricular dysfunction and atrial enlargement, potentially increaszing the risk of atrial fibrillation (AF) and heart failure.^7^ However, whether these effects differ significantly between SSS and AVB patients remains unclear.^8^ Furthermore, the clinical implications of pacemaker therapy, including rehospitalization rates, mortality, and symptomatic improvement, require further exploration.^9^

This study aims to compare the long-term clinical outcomes of patients undergoing pacemaker implantation for SSS versus AVB over a three-year follow-up period. By analyzing echocardiographic changes, pacemaker-recorded data, and clinical endpoints, we seek to provide a comprehensive assessment of the impact of pacing therapy on these two distinct patient populations. The findings from this study will contribute to a better understanding of the prognostic implications of pacemaker implantation and inform future strategies for patient management and therapy optimization.

## Methods

### Study design and population

This study was a retrospective, observational cohort study conducted to compare the long-term clinical outcomes of patients undergoing permanent pacemaker implantation for SSS or AVB. The study population included adult patients who underwent pacemaker implantation at our institution between January 2018 and December 2020. Inclusion criteria consisted of a confirmed diagnosis of either SSS or AVB as the primary indication for pacemaker implantation, age ≥18 years, and availability of baseline and follow-up clinical and echocardiographic data. Exclusion criteria included prior pacemaker implantation, concomitant severe valvular disease requiring intervention, advanced heart failure (New York Heart Association [NYHA] class IV), or a history of congenital heart disease.

### Data collection and follow-up

Demographic, clinical, laboratory, and echocardiographic data were collected from medical records. Baseline characteristics included age, sex, body mass index (BMI), comorbidities (hypertension, diabetes mellitus, coronary artery disease, and smoking status), and medication use (beta-blockers, ACE inhibitors/angiotensin receptor blockers [ARBs], mineralocorticoid receptor antagonists [MRAs], loop diuretics, and non-dihydropyridine calcium channel blockers). Laboratory parameters included serum sodium (Na), potassium (K), estimated glomerular filtration rate (eGFR), and hemoglobin levels.

### Echocardiographic assessment

All patients underwent transthoracic echocardiography at baseline and follow-up intervals (first month, sixth month, first year, and third year). Echocardiographic examinations were performed using a commercially available ultrasound system (Philips EPIQ 7) by experienced cardiologists following the American Society of Echocardiography (ASE) guidelines. Key parameters were measured as follows:

- *Left Ventricular Ejection Fraction (EF%):* Assessed using the biplane Simpson’s method from the apical four-chamber and two-chamber views.
- *Left Atrial Diameter (LAD):* Measured in the parasternal long-axis view at end-systole.
- *Left Atrial Volume Index (LAVI):* Calculated using the biplane area-length method and indexed to body surface area.
- *Left Ventricular End-Diastolic Diameter (LVEDD):* Measured at the level of the mitral valve leaflet tips in the parasternal long-axis view.
- *Pulmonary Artery Systolic Pressure (PASP):* Estimated using the peak tricuspid regurgitation velocity and right atrial pressure estimation.
- *Mitral Regurgitation (MR) Severity:* Assessed using color Doppler imaging and graded as mild, moderate, or severe based on the regurgitant jet area and vena contracta width.

### Pacemaker data collection and interpretation

Pacemaker programming data were obtained from device interrogation reports at each follow-up visit. The interrogation was performed using the Medtronic pacemaker programmer and involved assessment of:

- *Mean Lower Rate Limit:* The programmed minimum heart rate at which the pacemaker delivers pacing support.
- *Atrial Pacing Percentage:* The proportion of time atrial pacing is delivered, indicating the pacemaker's reliance on artificial pacing to maintain rhythm.
- *Atrial Premature Beat Percentage:* The percentage of premature atrial contractions detected, which may indicate underlying atrial arrhythmia.
- *Atrial High-Rate Episodes (AHREs):* Episodes of rapid atrial activity detected by the pacemaker, defined as atrial rates exceeding a predefined threshold (e.g., 180 beats per minute), which may suggest AF or atrial tachyarrhythmia.
- *Pacemaker Programming Protocol:* All pacemakers were programmed according to contemporary guidelines with optimization for physiologic pacing. The pacing mode was DDD/DDDR in all patients, with a lower rate limit of 60 beats per minute. In SSS patients, algorithms to minimize ventricular pacing were enabled when available, including extended AV delay (up to 250-300 ms) and AV hysteresis features to promote intrinsic AV conduction. However, in AVB patients, such algorithms were not utilized, given the intrinsic conduction disease, and standard AV delays (120-180 ms) were programmed. Rate-responsive pacing was activated in patients with chronotropic incompetence. Mode-switch algorithms for atrial tachyarrhythmias were enabled in all devices. Despite these programming strategies, the ventricular pacing burden in AVB patients remained high (>90%) due to the nature of their conduction disorder, whereas SSS patients maintained predominantly intrinsic ventricular activation.

### Outcome measures

The primary outcome was the longitudinal change in echocardiographic parameters over the three-year follow-up period. Secondary outcomes included:

- *Symptomatic improvement, assessed using the European Heart Rhythm Association (EHRA) symptom score:* The EHRA score is a classification system that evaluates the severity of symptoms related to arrhythmia, ranging from EHRA I (no symptoms) to EHRA IV (severe symptoms affecting daily activities).
- *Incidence of new-onset atrial fibrillation (AF):* Defined as the first documented episode of AF detected by either routine electrocardiography, Holter monitoring, or pacemaker interrogation, in a patient with no prior history of AF.
- *Rehospitalization rates during the follow-up period.*
- *All-cause mortality.*

### Statistical analysis

Continuous variables were expressed as mean ± standard deviation (SD) and compared between groups using the independent samples t-test. Categorical variables were presented as frequencies and percentages and analyzed using the chi-square or Fisher's exact test. Repeated measures analysis of variance (ANOVA) was used to assess changes in echocardiographic parameters over time. Cox proportional hazards regression analysis was performed to identify predictors of rehospitalization and mortality. Kaplan-Meier survival curves were generated for rehospitalization and mortality outcomes, and comparisons between groups were conducted using the Log-Rank test. A two-sided p-value <0.05 was considered statistically significant.

### Ethical considerations

This study was conducted in accordance with the principles of the Declaration of Helsinki and was approved by the institutional ethics committee. Given the retrospective nature of the study, informed consent was waived by the ethics board. Patient confidentiality was maintained by de-identifying data before analysis.

## Result

The study included a total of 192 patients, with 65 (33.9%) undergoing pacemaker implantation for SSS and 127 (66.1%) for AVB. The mean age of the cohort was 66.3 ± 7.2 years, and 89 patients (46.4%) were male. Hypertension was the most prevalent comorbidity, affecting 93 patients (48.4%).

The two groups were compared in terms of demographic, echocardiographic, and clinical characteristics. No significant differences were observed in age, sex, or BMI. The prevalence of hypertension, diabetes mellitus, coronary artery disease, and active smoking was similar between the groups; however, heart failure was more frequent in AVB patients than in those with SSS (17.3% vs. 10.8%, p=0.03). The use of ACE inhibitors/ARBs and mineralocorticoid receptor antagonists (MRAs) did not differ between groups. However, beta-blockers (69.2% vs. 13.4%, p<0.001) and non-dihydropyridine calcium channel blockers (12.3% vs. 7.1%, p=0.01) were more commonly prescribed in SSS patients. Conversely, loop diuretics were used more frequently in the AVB group (20.5% vs. 9.2%, p=0.05). Serum sodium (Na), potassium (K), estimated glomerular filtration rate (eGFR), and hemoglobin levels were comparable between the two groups. On echocardiographic assessment, LAD was significantly larger in AVB patients (34.6 ± 6.5 mm vs. 31.3 ± 6.9 mm, p=0.02), whereas no other significant differences were observed in the remaining parameters (Table 1).

**Table 1:** – Characteristics of the patients, including demographics, echocardiographic data, and clinical features

Patient characteristics	All patients (N=192)	Patients with sick sinus syndrome (N=65)	Patients with atrioventricular block (N=127)	p value
Age (years)	66.3±7.2	66.4±7.8	66.3±6.9	0.96
Gender (male)	89 (46.4)	28 (43.1)	61 (48.0)	0.52
BMI (kg/m^2^)	27.4±4.4	27.7±4.8	27.2±4.2	0.43
Hypertension	93 (48.4)	34 (52.3)	59 (46.5)	0.44
DM	34 (17.7)	12 (18.5)	22 (17.3)	0.85
Heart failure	29 (15.1)	7 (10.8)	22 (17.3)	0.03
Coronary artery disease	59 (30.7)	25 (38.5)	34 (26.8)	0.10
Active smoker	94 (49.0)	34 (52.3)	60 (47.2)	0.32
Beta blocker	62 (32.3)	45 (69.2)	17 (13.4)	<0.001
Nondihydropyridine CCB	17 (8.9)	8 (12.3)	9 (7.1)	0.01
ACEi/ARB	103 (53.6)	36 (55.4)	67 (52.8)	0.73
MRA	38 (19.8)	13 (20.0)	25 (19.7)	0.96
Loop diuretic	32 (16.7)	6 (9.2)	26 (20.5)	0.05
Na (mEq/L)	134.4±1.7	133.9±2.0	135.1±1.6	0.81
K (mmol/L)	4.3±1.1	4.4±1.0	4.3±1.2	0.89
eGFR (mL/min/1.73m^2^)	91.3±18.7	91.6±18.5	91.1±18.9	0.84
Hemoglobin (mg/dL)	13.7±1.9	13.6±2.0	13.7±1.8	0.85
EF%	56.1±6.7	57.2±6.0	55.5±7.0	0.11
LAD	33.5±6.8	31.3±6.9	34.6±6.5	0.02
LAVI	45.6±9.7	46.1±10.8	45.3±9.1	0.58
LVEDD	44.9±3.4	45.2±3.5	44.7±3.4	0.28
MR	56 (29.1)	17 (26.2)	39 (30.7)	0.66
PASP	26.2±6.7	26.9±6.2	25.8±7.0	0.30

ACEi/ARB: Angiotensin converting enzyme inhibitors/Angiotensin II receptor blockers; BMI: Body mass index; CCB: Calcium channel blocker; DM: Diabetes mellitus; EF: Ejection fraction; GFR: Glomerular filtration rate; K: Potassium; LAD: Left atrial diameter; LAVI: Left atrial volume index; LVEDD: Left ventricular end diastolic diameter; MR: Mitral regurgitation; MRA: Mineralocorticoid receptor antagonist; Na: Sodium; PASP: Pulmonary artery systolic pressure. *Values are mean ± SD, median (IQR) or n [n/N if missing data] (%). *Interquartile range [25% percentile-75% percentile].

The two patient groups were compared using pacemaker-recorded data. The mean lower rate limit at baseline did not differ significantly between groups (60.5 ± 2.5 vs. 61.2 ± 3.6, p = 0.20). As expected, atrial pacing percentage was consistently higher in the SSS group at all follow-up intervals (first month: 84.5±19.1% vs. 18.1±14.5%; third year: 88.4±20.3% vs. 17.3±21.3%; p < 0.001), while ventricular pacing percentage was markedly higher in AVB patients (first month: 92.5±6.3% vs. 12.3±8.5%; third year: 95.3±4.8% vs. 9.8±7.6%; p < 0.001). The minimal ventricular pacing in SSS patients reflects preserved AV conduction in this population, whereas the high ventricular pacing burden in AVB patients was inherent to their conduction disorder. Atrial premature beat percentage and atrial high-rate episode frequency were comparable between the groups throughout the follow-up period (Table 2).

**Table 2:** – Comparison of pacemaker data between patient groups during the follow-up period

Pacemaker data	Patients with sick sinus syndrome	Patients with atrioventricular block	p value
**Mean pacemaker lower rate limit (bpm)**	60.5±2.5	61.2±3.6	0.20
**Atrial pacing percentage (%)**			
First month	84.5±19.1	18.1±14.5	<0.001
Sixth month	86.0±19.4	15.2±20.0	<0.001
First year	87.2±22.2	16.8±22.6	<0.001
Third year	88.4±20.3	17.3±21.3	<0.001
**Ventricular pacing (%)**			
First month	12.3±8.5	92.5±6.3	<0.001
Sixth month	11.8±9.2	93.1±5.8	<0.001
First year	10.5±8.9	94.2±5.1	<0.001
Third year	9.8±7.6	95.3±4.8	<0.001
**Atrial premature beat percentage (%)**			
First month	5.9±2.1	6.4±1.9	0.57
Sixth month	5.9±1.7	6.3±1.4	0.66
First year	4.1±1.5	3.7±1.2	0.45
Third year	3.0±1.1	2.9±1.1	0.77
**Atrial High-Rate episode (%)**			
First month	9.9±1.8	10.3±4.1	0.88
Sixth month	9.9±3.9	10.1±3.2	0.97
First year	14±3.2	13.9±4.3	0.98
Third year	17.2±1.6	15.1±2.9	0.70

bpm: Beats per minute. *Values are mean ± SD, median (IQR) or n [n/N if missing data] (%). *Interquartile range [25% percentile-75% percentile].

Longitudinal changes in echocardiographic parameters were assessed from pre-implantation to 3-year follow-up in both patient subgroups. Among patients with SSS, we observed significant deterioration in multiple cardiac parameters. Left ventricular systolic function declined, with EF decreasing from 57.2 ± 6.0% at baseline to 54.3 ± 8.3% at 3 years (p < 0.001). These patients exhibited progressive left atrial remodeling, demonstrated by increases in LAD from 31.3 ± 6.9 mm to 38.8 ± 7.9 mm (p = 0.040) and LAVI from 46.1 ± 10.8 mL/m^2^ to 53.2 ± 10.4 mL/m^2^ (p < 0.001). Furthermore, left ventricular dimensions expanded significantly, with LVEDD increasing from 45.2 ± 3.5 mm to 51.0 ± 3.5 mm (p < 0.001). Valvular function also worsened, with the prevalence of mild or greater MR increasing substantially from 26.2% (17/65 patients) to 58.4% (38/65 patients; p = 0.001). Additionally, PASP rose from 26.9 ± 6.2 mmHg to 31.6 ± 7.0 mmHg (p=0.025). Patients with AVB similarly demonstrated a significant reduction in EF% from 55.5 ± 7.0% to 53.0 ± 6.8% (p < 0.001). However, in contrast to the SSS group, AVB patients maintained stable left atrial dimensions (LAD and LAVI) and LVEDD throughout the follow-up period. Both groups showed comparable progression of valvular dysfunction, with AVB patients exhibiting an increase in mild or worse MR from 30.7% (39/127 patients) to 57.4% (73/127 patients; p < 0.001) and a rise in PASP from 25.8 ± 7.0 mmHg to 30.8 ± 8.9 mmHg (p = 0.019) (Table 3).

**Table 3:** – Comparison of echocardiographic data between patient groups at baseline and 3-year follow-up

Echocardiographic data	Sick sinus syndrome			Atrioventricular block		
	Before PM	3rd year after PM	p value	Before PM	3rd year after PM	p value
EF%	57.2±6.0	54.3±8.3	<0.001	55.5±7.0	53.0±6.8	<0.001
LAD	31.3±6.9	38.8±7.9	0.040	34.6±6.5	36.3±7.4	0.10
LAVI	46.1±10.8	53.2±10.4	<0.001	45.3±9.1	48.4±9.2	0.14
LVEDD	45.2±3.5	51.0±3.5	<0.001	44.7±3.4	50.3±3.2	0.048
MR	17 (26.2)	38 (58.4)	0.001	39 (30.7)	73 (57.4)	<0.001
PASP	26.9±6.2	31.6±7.0	0.025	25.8±7.0	30.8±8.9	0.019

EF: Ejection fraction; LAD: Left atrial diameter; LAVI: Left atrial volume index; LVEDD: Left ventricular end diastolic diameter; MR: Mitral regurgitation; PASP: Pulmonary artery systolic pressure. *Values are mean ± SD, median (IQR) or n [n/N if missing data] (%). *Interquartile range [25% percentile-75% percentile]

At the 3-year follow-up assessment, clinical outcomes were evaluated according to EHRA symptom scores. The analysis revealed that 83.9% of patients (161 of 192) experienced symptomatic improvement following pacemaker implantation. When stratified by indication, 85.6% of patients with SSS (55 of 65) and 83.5% of those with AVB (106 of 127) demonstrated clinical benefit, with no statistically significant difference between groups (p=0.84; odds ratio [OR] 0.92, 95% confidence interval [CI] 0.40–2.09). Subsequent analysis of patient characteristics associated with symptomatic improvement yielded distinct patterns for each group. Among patients with SSS, no significant predictors of treatment response were identified. However, in the AVB cohort, several factors showed significant inverse correlations with symptomatic benefit: female sex (p=0.039), pre-existing coronary artery disease (p=0.006), increased LAD (p=0.05), and greater LVEDD (p=0.042). During the 3-year observation period, new-onset AF was documented in 38.5% of the study population (74 of 192). The incidence was comparable between etiological groups, occurring in 41.5% of SSS patients (27 of 65) versus 37.0% of AVB patients (47 of 127) (p=0.54; OR 0.83, 95% CI 0.45–1.52). Examination of potential risk factors for atrial fibrillation development revealed no significant associations in the SSS group. Conversely, among patients with AVB, three parameters emerged as significant predictors of AF occurrence: advanced age (p=0.045), larger LAD (p=0.003), and increased LAVI (p=0.046) (Figure 1).

**Figure 1:** – Comparison of EHRA symptom scale and development of atrial fibrillation between patient groups at 3-year follow-up.

At the 3-year follow-up evaluation, we assessed rehospitalization and mortality outcomes across the study cohort. Among the 192 enrolled patients, 107 (55.7%) required rehospitalization during the observation period. When stratified by pacemaker indication, rehospitalization rates were comparable between groups, occurring in 50.8% of SSS patients (33/65) versus 58.3% of AVB patients (74/127) (p=0.38; odds ratio [OR] 1.20, 95% confidence interval [CI] 0.80-1.81).

Predictive analysis of rehospitalization risk factors revealed distinct patterns between etiologies. For SSS patients, only elevated PASP demonstrated a significant association with rehospitalization (p=0.04). In contrast, the AVB cohort showed significant inverse relationships between rehospitalization and both female sex (p=0.037) and hypertension diagnosis (p=0.013). All-cause mortality was documented in 12.5% of patients (24/192), with no significant difference between groups (SSS: 13.8% [9/65] vs AVB: 11.8% [15/127]; p=0.69; OR 0.84, 95% CI 0.37-1.93). Mortality predictors differed substantially between groups. Among SSS patients, only the severity of MR showed significant correlation with mortality risk (p=0.031). Conversely, AVB patients demonstrated significant mortality associations with three independent factors: pre-existing coronary artery disease (p=0.009), beta-blocker therapy (p=0.005), and elevated LAVI (p=0.001) (Figure 2).

**Figure 2:** – Comparison of rehospitalization and mortality events between patient groups at 3-year follow-up.

During the 3-year follow-up, the groups were compared using Cox regression and survival analysis for rehospitalization and mortality outcomes. No significant difference was observed in mortality between groups (Log Rank p=0.688). However, survival analysis for rehospitalization revealed a trend toward lower rehospitalization rates over time in sick sinus syndrome patients, though this did not reach statistical significance (Log-Rank p=0.373) (Figure 3). Key comparative findings between the SSS and AVB groups regarding cardiac remodeling, clinical outcomes, pacing characteristics, and mortality predictors are summarized in the Central Illustration.

**Figure 3:** – Log-Rank analysis of rehospitalization and mortality events at 3-year follow-up.

## Discussion

This study provides a comprehensive comparison of the long-term clinical outcomes between patients with SSS and AVB following permanent pacemaker implantation. Our findings demonstrate that while pacemaker therapy provides symptomatic improvement in the majority of patients from both groups, important differences exist in cardiac remodeling patterns and predictors of adverse outcomes.

The observed higher atrial pacing percentage in SSS patients is consistent with underlying sinus node dysfunction, which necessitates more frequent atrial pacing support. ^4,10^ This finding aligns with previous studies that have demonstrated the dependence of SSS patients on atrial pacing for maintaining adequate heart rates.^11^ In contrast, AVB patients typically maintain intrinsic sinus node function but require ventricular pacing due to impaired AV conduction.^5^

The substantially different ventricular pacing burdens between groups (>90% in AVB vs. <12% in SSS patients) provide crucial context for interpreting our remodeling findings. The high ventricular pacing percentage in AVB patients (averaging 93-95% throughout follow-up) likely contributed to the adverse effects observed in both groups, though the patterns differed. While both groups experienced declining EF, the progressive left atrial and ventricular dilation observed predominantly in SSS patients despite minimal ventricular pacing suggests that sinus node dysfunction itself may predispose to adverse remodeling independent of pacing burden. Conversely, the stable chamber dimensions in AVB patients despite near-universal ventricular pacing challenges the notion that RV pacing universally causes chamber dilation, suggesting other factors, such as intrinsic atrial disease in SSS, may play important roles. These findings align with recent observations that the detrimental effects of RV pacing may be more pronounced in patients with underlying atrial pathology.

A particularly notable finding was the differential pattern of cardiac remodeling between the two groups. SSS patients exhibited significant increases in left atrial dimensions and LVEDD over the follow-up period, suggesting progressive adverse cardiac remodeling. This observation supports earlier studies suggesting that right ventricular pacing might contribute to ventricular dyssynchrony and subsequent adverse remodeling.^7,12^ The maintenance of stable left atrial dimensions in AVB patients, despite comparable decreases in EF, suggests potential differences in the pathophysiological response to pacing therapy between these two conditions.

The substantial incidence of new-onset AF in both groups (41.5% in SSS vs. 37.0% in AVB) highlights the close relationship between bradyarrhythmia and AF development.^13^ While the difference between groups was not statistically significant, the identification of advanced age and increased left atrial dimensions as predictors of AF in AVB patients aligns with established risk factors for this arrhythmia.^14^ The high incidence of AF raises important questions about the potential benefits of prophylactic anticoagulation in high-risk patients with pacemakers, particularly those with increased left atrial dimensions.^15^

Regarding symptomatic improvement, our finding that approximately 84% of patients experienced clinical benefit following pacemaker implantation confirms the efficacy of pacing therapy for symptom relief in bradyarrhythmias.^16^ The absence of significant between-group differences in symptomatic improvement suggests that the underlying etiology of bradyarrhythmia may not substantially influence the subjective response to pacing therapy. However, the identification of female sex, coronary artery disease, and increased cardiac dimensions as negative predictors of symptomatic improvement in AVB patients warrants further investigation and may have implications for patient selection and counseling.^17^

The rehospitalization rate of approximately 56% over three years indicates a substantial healthcare burden associated with pacemaker patients. Although the trend toward lower rehospitalization rates in SSS patients did not reach statistical significance, the different predictors of rehospitalization between groups suggest potentially distinct pathophysiological mechanisms contributing to clinical decompensation.^18^ The association between elevated PASP and rehospitalization in SSS patients points to the potential importance of right ventricular function and pulmonary hemodynamics in this population.^19^

Mortality outcomes were comparable between groups, affecting approximately 12% of patients over three years. However, the distinct predictors of mortality—MR severity in SSS patients versus coronary artery disease, beta-blocker therapy, and increased left atrial volume in AVB patients—suggest different pathways contributing to adverse outcomes.^20,21^ The association between beta-blocker therapy and mortality in AVB patients is particularly intriguing and may reflect either the severity of underlying cardiac disease necessitating beta-blocker use or potentially adverse interactions between pharmacological therapy and pacing.^22^

The progressive increase in MR observed in both groups deserves special attention. This finding is consistent with previous studies suggesting that right ventricular pacing can induce papillary muscle dyssynchrony and subsequent functional MR.^23^ The correlation between MR severity and mortality in SSS patients emphasizes the potential prognostic importance of valvular function in this population.^24^

This study provides several important advances beyond existing literature. First, we demonstrate for the first time that SSS patients exhibit progressive cardiac remodeling despite minimal ventricular pacing (<12%), contradicting the prevailing assumption that adverse remodeling is primarily pacing-induced. This finding suggests an intrinsic pathophysiological process in SSS that warrants recognition as an independent risk factor for structural deterioration, not previously emphasized in major pacemaker trials such as MOST or DAVID. Second, our observation that AVB patients maintain stable chamber dimensions despite >90% ventricular pacing challenges the universality of pacing-induced cardiomyopathy and suggests protective factors or compensatory mechanisms that merit further investigation. Third, we identify etiology-specific mortality predictors that have not been previously described: while MR predicts outcomes in SSS (potentially related to progressive atrial dysfunction), the predictive value of LAVI in AVB patients suggests different mechanistic pathways to adverse outcomes. These distinct risk profiles provide a rationale for differentiated surveillance strategies based on underlying bradyarrhythmia etiology—a precision medicine approach not currently reflected in guideline recommendations, which treat all pacemaker patients similarly regardless of indication. Finally, the high incidence of new-onset AF (41.5% in SSS, 37.0% in AVB) substantially exceeds rates reported in earlier trials, likely reflecting both improved detection through continuous device monitoring and our longer follow-up duration, emphasizing the need for systematic atrial arrhythmia surveillance in all pacemaker recipients.

Our study has several limitations that should be acknowledged. First, its retrospective design limits causal inference and may introduce selection bias. Second, we did not systematically assess ventricular dyssynchrony, which might have provided mechanistic insights into the observed cardiac remodeling patterns. Third, the single-center nature of our study may limit generalizability to other populations. Finally, we did not analyze the impact of different pacing modes or settings, which might influence long-term outcomes.

Despite these limitations, our findings have important clinical implications. The different patterns of cardiac remodeling and predictors of adverse outcomes between SSS and AVB patients suggest that individualized follow-up strategies based on the underlying etiology of bradyarrhythmia may be beneficial. SSS patients may benefit from closer monitoring of cardiac dimensions and optimization of pacing settings to minimize ventricular pacing burden.^25^ In AVB patients, particular attention to left atrial parameters and management of underlying coronary artery disease might improve long-term outcomes.^26^

Future research should explore whether alternative pacing strategies, such as His bundle pacing or cardiac resynchronization therapy, might prevent the observed structural changes and improve outcomes in specific patient subgroups.^27,28^ Additionally, prospective studies with longer follow-up periods are needed to better understand the long-term impact of pacing therapy on cardiac structure and function, particularly in relation to the development of heart failure and valvular dysfunction.^29^

## Conclusion

This three-year comparative study demonstrates that while permanent pacemaker implantation provides comparable symptomatic improvement in both SSS and AVB patients, the two conditions exhibit fundamentally different pathophysiological responses to pacing therapy. Despite minimal ventricular pacing burden, SSS patients developed progressive adverse cardiac remodeling with significant left atrial and ventricular chamber dilation, suggesting that sinus node dysfunction itself, independent of pacing, predisposes to structural deterioration. Conversely, AVB patients maintained stable chamber dimensions despite near-universal ventricular pacing (>90%), challenging conventional assumptions about the uniformly deleterious effects of right ventricular pacing.

The distinct mortality predictors between groups, MR severity in SSS versus coronary artery disease, and left atrial volume in AVB underscore the need for etiology-specific risk stratification and tailored follow-up strategies. These findings support individualized management approaches: closer echocardiographic surveillance for structural changes in SSS patients, and enhanced attention to left atrial parameters and coronary disease management in AVB patients. Future research should investigate whether alternative pacing strategies can mitigate the intrinsic remodeling tendency observed in SSS patients and validate risk-based surveillance protocols for each patient population.
